# The complete mitochondrial genome of the lesser aspen webworm moth *Meroptera pravella* (Insecta: Lepidoptera: Pyralidae)

**DOI:** 10.1080/23802359.2017.1334525

**Published:** 2017-06-05

**Authors:** Living Prairie Mitogenomics Consortium

**Affiliations:** Department of Biological Sciences, University of Manitoba, Winnipeg, MB, Canada

**Keywords:** Illumina sequencing, mitogenomics, inquiry-based learning, Pyraloidea, Pyralidae

## Abstract

The lesser aspen webworm moth, *Meroptera pravella*, is a small pyralid that uses quaking aspen (*Populus tremuloides*) and related tree species as larval hosts. Whole-genome Illumina sequencing allowed the assembly of a complete circular mitochondrial genome of 15,260 bp consisting of 80.7% AT nucleotides, 22 tRNAs, 13 protein-coding genes, 2 rRNAs and a control region. Mitogenome structure maintains complete synteny with other sequenced pyralid mitogenomes. Parsimony and maximum-likelihood phylogenetic reconstruction places *M. pravella* within monophyletic subfamily Phycitinae and monophyletic family Pyralidae. The Pyralidae, with monophyletic sister family Crambidae, constitute monophyletic superfamily Pyraloidea, which is consistent with conventional taxonomy.

The Living Prairie Mitogenomics Consortium seeks to accumulate arthropod mitochondrial genomes from a single location to produce a reference library for improved DNA-based species identification and phylogenetics (McCullagh 2016). Mitochondrial genome sequences were assembled and annotated by undergraduates in a course of inquiry-based learning exercise (Marcus et al. 2010). Students analyzing the data successfully (which were further curated by the instructor) belong to our consortium.

The Living Prairie Museum (LPM) consists of 12.9 hectares of relict unploughed prairie maintained by periodic controlled burns located in Winnipeg, Manitoba, Canada (GPS 49.889607 N, −97.270487 W). Over 160 native plant species occur at LPM, supporting a rich arthropod fauna. Arthropods were sampled weekly during the 2015 growing season.

On 17–18 July 2015, a USDA blacklight trap (Winter 2000) was deployed to collect night-flying insects. One adult specimen of the lesser aspen webworm moth *Meroptera pravella* (Pyralidae, project specimen number 2015.07.17.012) was trapped. This specimen likely originated from a nearby grove of quaking aspen (*Populus tremuloides*) larval host plant located at LPM. The specimen was pinned, spread and deposited in the collection of the Wallis Roughley Museum of Entomology at the University of Manitoba (voucher JBWM0363025).

DNA was prepared (McCullagh & Marcus 2015) and sequenced by Illumina MiSeq (San Diego, CA) (Peters & Marcus 2017). Overall, 2,958,411 paired reads (total 1.7 Gb) were assembled in Geneious 10.1.2 to a *Plodia interpunctella* (Pyralidae) reference mitogenome (KT207942.1) to reconstruct a complete mitogenome sequence for *M. pravella* (GenBank MF073207). Annotation was performed with reference to *P. interpunctella* and *Junonia lemonias* (Nymphalidae, KP941756) mitogenomes (McCullagh & Marcus 2015). The complete *M. pravella* nuclear rRNA repeat (GenBank MF073208) was also assembled and annotated with respect to the *Attacus ricini* rRNA repeat (Saturniidae, AF463459)

The circular mitogenome of *M. pravella* consists of 15,260 bp with nucleotide composition of 39.5% A, 11.6% C, 7.7% G and 41.0% T. *Meroptera pravella* maintains complete synteny with other mitogenomes from superfamily Pyraloidea and other Ditrysian Lepidoptera (Cao et al. 2012). *Meroptera pravella COI* has an aberrant start codon (CGA) that is typical of insects (Peters & Marcus 2016). Four mitochondrial protein-coding genes (*NAD2, COI, COII* and *NAD5*) have aberrant single-nucleotide (T) stop codons. As in many other arthropods, all *M. pravella* tRNAs have standard cloverleaf secondary structures except for trnS (AGN) which has the dihydrouridine arm replaced by a loop (McCullagh & Marcus 2015). The rRNAs (775 bp 12S and 1382 bp 16s) are composed of 84.7% AT while the putative control region (278 bp) is 96.0% AT.

We reconstructed a phylogeny using mitogenomes from *M. pravella*, 7 other Pyralid moth species, 24 Crambidae species and representatives from the related families Thyrididae, Alucitidae and Pterophoridae. Sequences were aligned in CLUSTAL Omega (Sievers et al. 2011) and analyzed by parsimony and maximum likelihood in PAUP* 4.0b8/4.0d78 (Swofford 2002) (Figure 1). Phylogenetic analysis places *M. pravella* as the basal lineage within subfamily Phycitinae of family Pyralidae and supports the conventional sister taxon relationship between monophyletic families Pyralidae and Crambidae in superfamily Pyraloidea (Munroe & Solis 1998).

**Figure 1.**
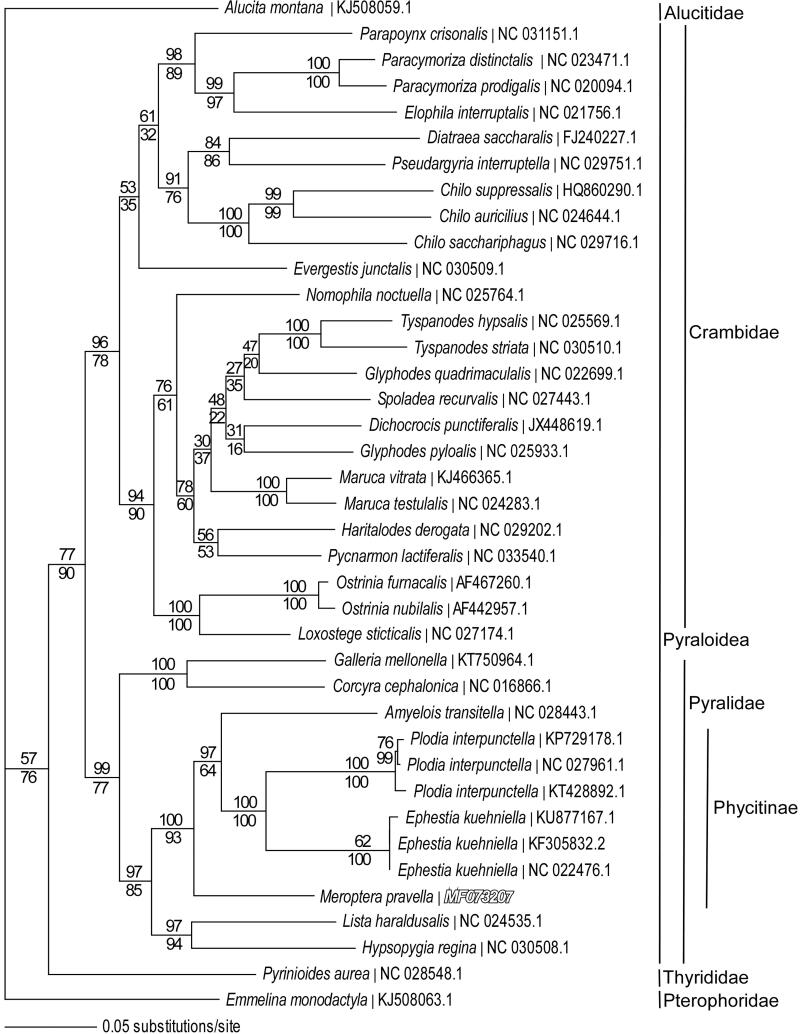
Maximum-likelihood phylogeny (GTR + I + G model, I = 0.2790, G = 0.4760, likelihood score 190,748.88) of *Meroptera pravella* and related species in families Pyralidae, Crambidae, Thyrididae, Alucitidae and Pterophoridae based on one million random addition heuristic search replicates (with tree bisection and reconnection) of aligned complete mitochondrial genomes. One million maximum parsimony heuristic search replicates produced a nearly identical tree topology for family Pyralidae (parsimony score 41,119 steps), but with a monophyletic *Glyphodes* and with *Evergestis* as sister to the Diatraea-Pseudargyria-Chilo clade in the Crambidae. Numbers above each node are maximum-likelihood bootstrap values and numbers below each node are maximum parsimony bootstrap values (each from one million random fast addition search replicates).

## References

[CIT0001] Cao YQ, Ma C, Chen JY, Yang DR. 2012 The complete mitochondrial genomes of two ghost moths, *Thitarodes renzhiensis* and *Thitarodes yunnanensis*: the ancestral gene arrangement in lepidoptera. BMC Genom. 13:276. 10.1186/1471-2164-13-276 PMC3463433 22726496

[CIT0002] Marcus JM, Hughes TM, McElroy DM, Wyatt RE. 2010 Engaging first year undergraduates in hands-on research experiences: the Upper Green River Barcode of Life Project. J Coll Sci Teach. 39:39–45.

[CIT0003] McCullagh BS, Marcus JM. 2015 The complete mitochondrional genome of Lemon Pansy, *Junonia lemonias* (Lepidoptera: Nymphalidae: Nymphalinae). J Asia-Pacific Ent. 18:749–755.

[CIT0004] McCullagh BS. 2016 Sequence evolution among divergent mitochondrial haplotypes within species of Junonia butterflies. M.Sc. Thesis. Winnipeg (MB) Canada: Department of Biological Sciences, University of Manitoba.

[CIT0005] Munroe EG, Solis MA. 1998 The pyraloidea In: Kristensen NP, editor. Lepidoptera, moths and butterflies volume 1: evolution, systematics, and biogeography. Berlin (Germany): Walter de Gruyter; p. 233–256.

[CIT0006] Peters MJ, Marcus JM. 2016 The complete mitochondrial genome of the Bermuda buckeye butterfly *Junonia coenia bergi* (Insecta: Lepidoptera: Nymphalidae). Mitochondrial DNA B. 1:739–741. 10.1080/23802359.2016.1159929 PMC7800380 33490418

[CIT0007] Peters MJ, Marcus JM. 2017 Taxonomy as a hypothesis: testing the status of the Bermuda buckeye butterfly *Junonia coenia bergi* (Lepidoptera: Nymphalidae). Syst Ent. 42:288–300.

[CIT0008] Sievers F, Wilm A, Dineen D, Gibson TJ, Karplus K, Li W, Lopez R, McWilliam H, Remmert M, Soding J, et al 2011 Fast, scalable generation of high-quality protein multiple sequence alignments using Clustal Omega. Mol Syst Biol. 7:539. 21988835 10.1038/msb.2011.75 PMC3261699

[CIT0009] Swofford DL. 2002. PAUP*. Phylogenetic Analysis Using Parsimony (*and Other Methods). Version 4. Sinauer Associates; Sunderland, MA.

[CIT0010] Winter WD. 2000 Basic techniques for observing and studying moths and butterflies. Vol. 5 Los Angeles (CA): The Lepidopterists' Society.

